# Mannose synergizes with chemoradiotherapy to cure cancer via metabolically targeting HIF‐1 in a novel triple‐negative glioblastoma mouse model

**DOI:** 10.1002/ctm2.226

**Published:** 2020-11-08

**Authors:** Feng Liu, Xiaohong Xu, Chunyang Li, Chunyan Li, Yuanjun Li, Songlin Yin, Shangbin Yu, Xiao Qian Chen

**Affiliations:** ^1^ Department of Pathophysiology School of Basic Medicine Tongji Medical College Key Laboratory of Ministry of Education for Neurological Disorders Huazhong University of Science and Technology Wuhan China; ^2^ Department of Nuclear Medicine Union Hospital Tongji Medical College Hubei Province Key Laboratory of Molecular Imaging Huazhong University of Science and Technology Wuhan China

Dear Editor,

The standard care of glioblastoma multiforme (GBM), which is surgery followed by temozolomide concurrent radiotherapy (RT/TMZ), confers limited survival benefit for patients.^1^ Additionally, current animal models do not faithfully recapitulate human GBM features, impeding animal‐to‐human translation.^2^ Here, we developed and characterized a novel orthotopic murine GBM model, in which we tested the efficacy of the RT/TMZ supplemented with mannose (RT/TMZ/Man).

Following the optimized protocol illustrated in Figure 1A, we established a stable subcutaneous‐intracranial G422‐GBM syngeneic mouse model system. Subcutaneous injection of 1 × 10^6^ fresh G422 cells led to 100% tumor formation, and the tumors reaching 1 cm^3^ on day 7–9 postimplantation (p.i.) were used for further experiments (Figure 1B). Microinjection of 1 × 10^3^–1 × 10^5^ G422 cells in mouse striatum caused 100% lethality within 30 days (Figure 1C) and 5 × 10^4^ cells, which led to mouse death within 14–23 days (Figure S1A), were selected for establishing the orthotopic model. We then determined the pathology and molecular characters of G422‐tumors. The brain slices showed rapid G422‐tumor development during day 7–15 p.i. (Figure S1B). The G422‐tumors were GFAP^+^Vimentin^+^CD3^−^ and infiltrated into distant brain areas (Figure 1D‐G) on day 5 p.i. They were *IDH1/2* ^WT^chromosome1/19^Intact^ *TERT‐*promoter^WT^ with *ATRX* ^Mutant^ and *Trp53* ^Mutant^ (Figure 1H), determined by the whole genome sequencing, and thus belonged to the triple‐negative (TN) primary GBM subtype.^3^ Our model was therefore named the G422^TN^‐GBM.

**FIGURE 1:**
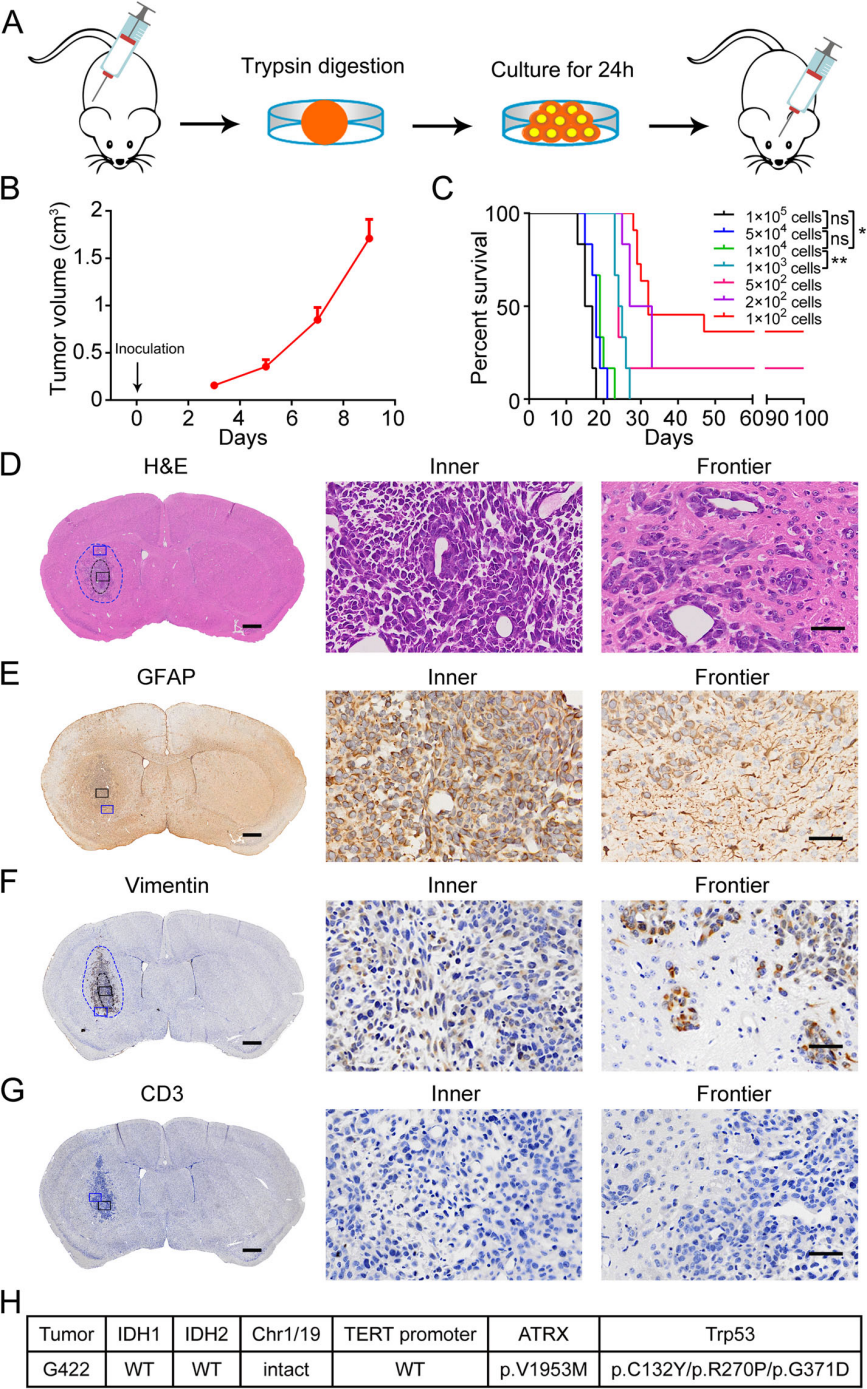
Establishment and characterization of the preclinical orthotopic mouse G422 glioma model. A, Schematic diagram of the process of establishing the stable orthotropic G422‐glioma murine model. 1 × 10^6^ G422 cells freshly isolated from subcutaneous tumors were used in the establishment of stable subcutaneous G422‐model. G422 tumors of 1 cm^3^ after 7–9 days of subcutaneous growth were used for subsequent experiments. After a short incubation of G422 cells in vitro, 5 × 10^4^ living G422 cells were microinjected into the right striatum of syngeneic mice to establish stable orthotopic G422‐model. B, The growth curve of subcutaneous G422 tumor (n = 6). C, The Kaplan‐Meier survivals of the orthotopic G422‐mice (n = 6‐11/group, * *P *< .05; ** *P *< .01; ns, not statistically significant). D‐G, H&E (D), GFAP (E), Vimentin (F), and CD3 (G) staining of the G422 glioma on day 5 postimplantation (p.i.). Black and blue dotted circles in the left panels delineate the tumor parenchyma and infiltrating boundary, respectively. Representative inner and frontier regions of tumor are, respectively, indicated by the black and blue rectangles in the left panels and their amplifications are shown in the middle and right panels, respectively. Scale bar, 50 μm. H, Genetic features of G422 glioma

Next, we determined the therapeutic responses of the orthotopic G422^TN^‐GBM to conventional surgery, radiotherapy, temozolomide, and the therapies of combining these two or three. Surgery alone, performed on day 7 p.i., effectively removed the tumor and slightly extended the median survival of the G422‐mice (Figure 2A‐C and Figure S2). A single dose (10 Gy) of whole brain irradiation did not enhance the animal survival, while the TMZ monotherapy started on day 7 p.i. significantly increased the survival (Figure 2D and E and Figure S3). RT/TMZ further improved the survival compared to either RT or TMZ monotherapy (RT/TMZ vs RT, *P *= .0002; RT/TMZ vs TMZ, *P *= .0166; Figure 2E). However, surgery did not offer extra survival benefit for the G422‐mice treated with the RT/TMZ (surgery/RT/TMZ vs RT/TMZ, *P *= .5770; Figure 2F). Therefore, the RT/TMZ treatment, without being combined with the surgical resection, was used for the following therapeutic studies.

**FIGURE 2:**
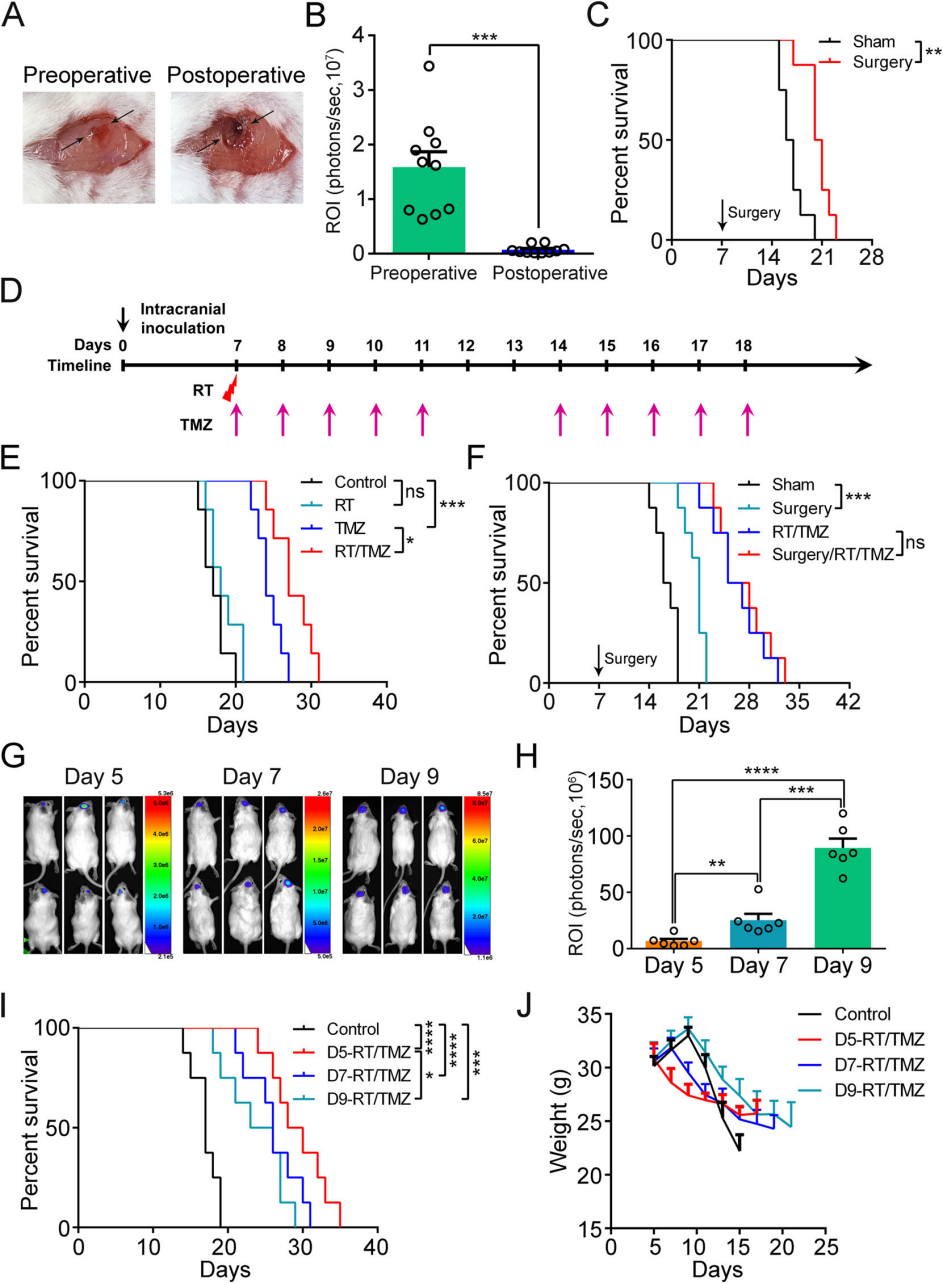
Therapeutic responses of the G422^TN^‐GBM model to the conventional surgery/RT/TMZ therapies. A, Preoperative and postoperative intracranial G422 tumor on day 7 p.i., imaged under dissection microscope. The opposite arrows mark the tumor location under the skull. B, Statistical analysis of bioluminescent ROI values of the pre‐ and postoperative tumors (n = 10). C, The Kaplan‐Meier survivals of the G422‐mice with or without receiving surgery (n = 8/group). D, Schematic diagram depicting the irradiation (RT), TMZ chemotherapy (TMZ), or their combined regimen started on day 7 p.i. RT, a single dose of 10 Gy‐whole brain irradiation (WBI); TMZ, 10 doses of TMZ during one therapeutic course with one oral gavage of 50 μg TMZ per gram of body weight in each dose. E, The Kaplan‐Meier survivals of the G422‐mice subjected to different treatments on day 7 as indicated (n = 7/group). F, The Kaplan‐Meier survivals of the G422‐mice subjected to the indicated treatments (n = 8/group). RT/TMZ, TMZ concurrent radiotherapy; Surgery/RT/TMZ, RT/TMZ preceded by surgery. G‐H, Representative bioluminescent images and statistical analysis of the ROI values of the intracranial tumors monitored on day 5, 7, and 9 p.i. (n = 6/group). I‐J, The Kaplan‐Meier survivals and body weight changes over time of the G422‐mice that received RT/TMZ started on the fifth (D5‐RT/TMZ), seventh (D7‐RT/TMZ), or ninth days (D9‐RT/TMZ) p.i. (n = 8/group) (*P < .05; **P < .01; ***P < .001; ****P < .0001; ns, not statistically significant)

In clinic, GBM patients diagnosed and treated early exhibit better therapeutic responses.^4^ Bioluminescent imaging and H&E staining showed progressive growth and infiltration of the G422 tumors on day 5, 7, and 9 p.i. (Figure 2G and H and Figure S4), which could represent the early, middle or late stages of GBM, respectively. The survivals of the G422‐mice that received the RT/TMZ regimen started on either day 5, 7, or 9 p.i. were all significantly improved compared to that of the control group (Figure 2I and J). Importantly, the RT/TMZ treatment started on day 5 was significantly more effective in extending animal survival compared to the one started on day 9 (*P *= .0147, Figure 2I). Nevertheless, with this most effective regimen that we had tested thus far, the G422‐mice still exhibited 100% lethality within 35 days p.i. Taken together, our G422^TN−^GBM model recapitulates the refractory character of human GBM to standard therapies in clinic, which is superior for therapeutic studies to our previous G422‐model.^5^

We next used the G422^TN−^GBM model as a preclinical tool to explore treatments for GBM. The monosaccharide mannose, found naturally in high amounts in many fruits, was recently highlighted as an effective anti‐cancer drug by impeding glucose metabolism.^6^ The PET/CT imaging with ^[18F]^FDG verified that oral administration of mannose indeed inhibited glucose metabolism of subcutaneous or intracranial G422 gliomas (Figure 3A and Figure S5). Mannose monotherapy started on day 5 p.i. slightly but significantly prolonged the survival of the G422‐mice as compared to the control and glucose‐treated groups (*P *= .0074 and .0282, respectively) (Figure 3B). In addition, mannose supplement significantly enhanced the therapeutic efficacy of both TMZ monotherapy (TMZ/Man vs TMZ, *P *= .0260) and RT/TMZ (RT/TMZ/Man vs RT/TMZ, *P *= .0085), but did not synergize with RT (RT/Man vs RT, *P *= .1100) (Figure 3C and Figure S6). Remarkably, 50% or 25% of G422‐mice that received the RT/TMZ/Man started on day 5 or 7 p.i. achieved long‐term survival (LTS), defined by animal survival of over 100 days, while none of the G422‐mice receiving RT/TMZ/Man on day 9 survived over 35 days (Figure 3C–E and Figure S6). In contrast to mannose, neither metformin^7^ nor disulfiram^8^ enhanced the RT/TMZ started on day 7 p.i. to achieve LTS for the G422‐mice (Figure S7). In summary, mannose can achieve disease cure in combination with RT/TMZ at the early and middle tumor developmental stages.

**FIGURE 3:**
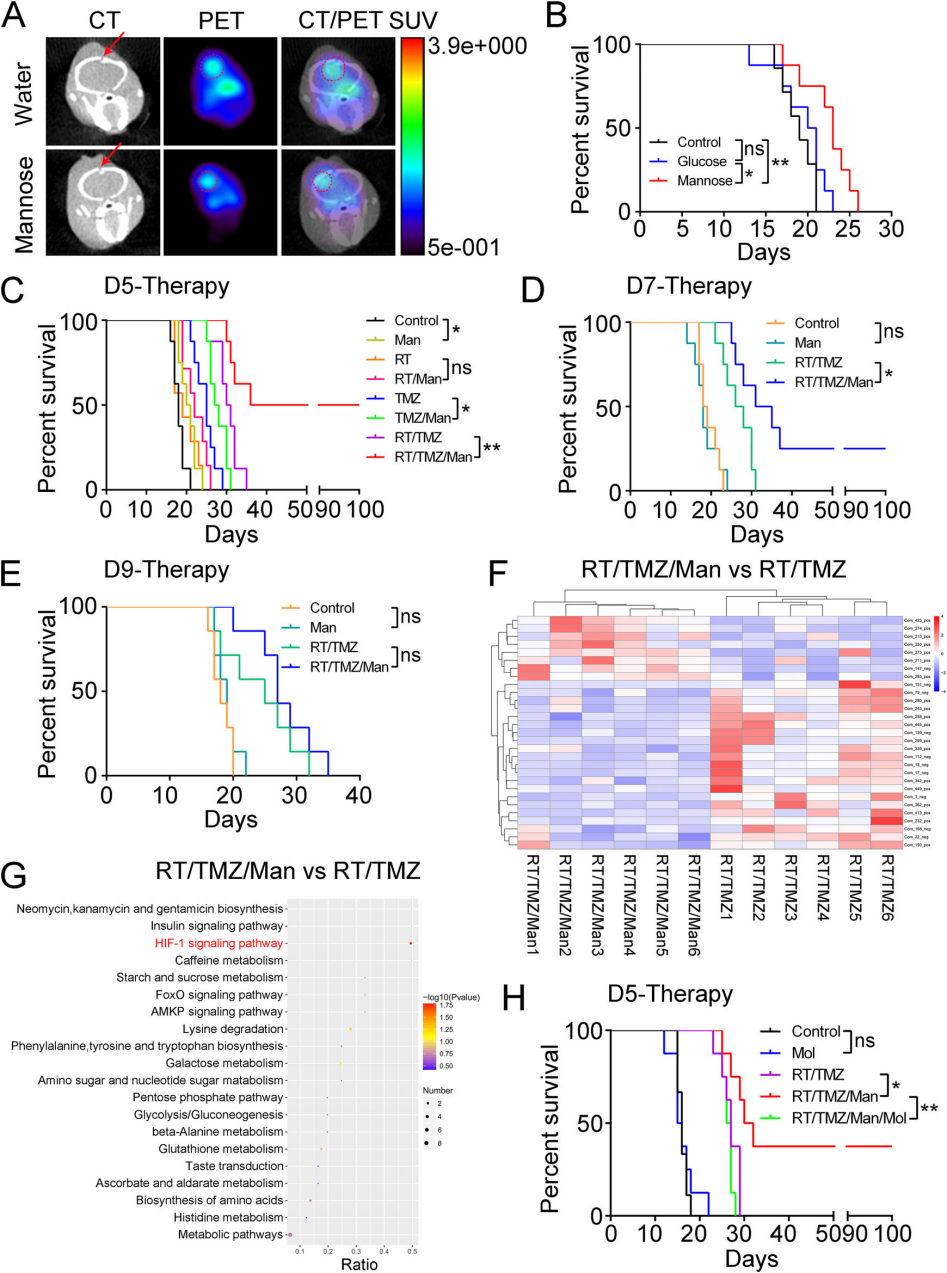
Mannose plus RT/TMZ achieves long‐term survival via metabolically suppressing HIF‐1 signaling pathway. A, Representative PET/CT imaging of intracranial G422 gliomas denoted by the red dotted circles. The red arrows indicate the tumor cell injection site. B, The Kaplan‐Meier survivals of the G422‐mice after treated with mannose or glucose (n = 7‐8/group). C‐E, The Kaplan‐Meier survivals of the G422‐mice treated with the indicated therapies started on day 5 (C), 7 (D), and 9 (E) p.i. (n = 7‐8/group). F, Clustered metabolite heat map showing significantly differential metabolites between the RT/TMZ/Man and RT/TMZ (n = 6/group). G, The top 20 differential pathways between the RT/TMZ/Man and RT/TMZ identified by the KEGG pathway enrichment analysis. Significantly altered pathways are highlighted in red fonts. H, The Kaplan‐Meier survival curves of the G422‐mice treated with the indicated therapies started on the fifth day (n = 8‐9/group) (*P < .05; **P < .01; ns, not statistically significant)

The RT/TMZ/Man therapy suppresses cell proliferation and invasion and increases DNA damage, as indicated by the decreased Ki‐67, vimentin/PD‐L1, and increased γ‐H2AX in the G422 tumors (Figure S8). Lastly, we explored the molecular mechanisms through which RT/TMZ/Man cures the G422‐mice by metabolomic analysis. The identified metabolites that are significantly differentially expressed between the RT/TMZ/Man and the RT/TMZ, Man, or untreated‐subcutaneous G422 gliomas were listed in the heat maps (Figure 3F and Figure S9). KEGG pathway enrichment analysis of these differential metabolites identified the HIF‐1 signaling pathway as the only significantly altered pathway between the RT/TMZ/Man and RT/TMZ groups (Figure 3G). Western blot assay verified the downregulation of HIF‐1α/VEGF and the increase of PHD2 that signals HIF degradation (Figure S10). Pharmacological restoration of HIF‐1α/VEGF by using the PHD2 inhibitor Molidustat (Mol) completely abolished the LTS endowed by RT/TMZ/Man (RT/TMZ/Man/Mol vs RT/TMZ/Man, *P *= .0037) and there was no statistical difference between the RT/TMZ and RT/TMZ/Man/Mol groups (*P *= .2045, Figure 3H and Figure S10). These results demonstrated the dominant role of the HIF‐1 pathway underlying the RT/TMZ/Man efficacy.

In conclusion, we established a highly reproducible syngeneic G422^TN^‐GBM mouse model that faithfully recapitulates the aggressiveness and therapeutic responses of human GBM, which still requires further verification. We discovered that the RT/TMZ/Man could offer disease cure for GBM in our model through metabolically abolishing the HIF‐1‐mediated resistance. Our work highlights a novel and superior preclinical mouse model for drug screening and the potential therapeutic value of the RT/TMZ/Man regimen for GBM.

## CONFLICT OF INTEREST

The authors declare that they have no conflict of interest.

## ETHICS APPROVAL AND CONSENT TO PARTICIPATE

All animal handling and experiments were performed in accordance with the NIH guidelines and approved by the Institutional Ethics Committees of Huazhong University of Science and Technology.

## AUTHOR CONTRIBUTIONS

Feng Liu performed experiments, analyzed data, and wrote the manuscript. Xiaohong Xu, Chunyang Li, and Songlin Yin assisted with animal experiments. Yuanjun Li and Chunyan Li assisted with PET/CT experiments. Shangbin Yu assisted with experimental design. Xiao Qian Chen designed the scheme of the study and wrote the manuscript.

## Supporting information

Supporting information
