# Supplementary material for: Mannose synergizes with chemoradiotherapy to cure cancer via metabolically targeting HIF‐1 in a novel triple‐negative glioblastoma mouse model
Source: Clin Transl Med. 2020 Nov 8;10(7):e226. doi: 10.1002/ctm2.226 (PMC7648968; doi:10.1002/ctm2.226)
Supplement: Supplementary file 1 — Supporting information [file CTM2-10-e226-s001.docx]

**Supplementary information**

**Mannose synergizes with chemoradiotherapy to cure cancer via metabolically targeting HIF-1 in a novel triple-negative glioblastoma mouse model**

Feng Liu, Xiaohong Xu, Chunyang Li, Chunyan Li, Yuanjun Li, Songlin Yin, Shangbin Yu, Xiao Qian Chen**Material and methods**

**Syngeneic mouse models of G422-GBM**

Adult male Kunming mice (18-22 g) were purchased from the Experimental Animal Centre, Huazhong University of Science and Technology (HUST). All animal handling and experiments were performed in accordance with the NIH guidelines and approved by the Institutional Ethics Committees of HUST. Kunming mice bearing subcutaneous G422 tumor (0.5 cm^3^) were purchased from the Cell Recourse Center, Institute of Basic Medical Sciences, Chinese Academy of Medical Sciences. The murine G422 glioma was originally derived from malignant astrocytoma formed in the brains after intracerebral implantation of 20-methylcholanthrene pellets into a total of 523 Kunming mice in 1964^1^. The tumor was then passaged in Kunming mice with serial intracranial-subcutaneous alternative transplantations. By nearly 10 years of serial transplantations, the G422 glioma has been passed on to the 120^th^ generations and became a stable histologically lower-differentiated GBM. The tumor cells were stored in liquid nitrogen.

For the establishment of syngeneic subcutaneous G422 mouse models, freshly isolated 1×10^6^ G422 in 0.2 ml PBS were inoculated into the right flank of Kunming mouse. The tumor growth was measured every other day. For the establishment of syngeneic orthotopic model, optimized subcutaneous G422 tumors (reached 1 cm^3^ at day 7-9 after inoculation) were used. Freshly isolated G422 cells were incubated in RPMI Medium 1640 basic supplemented with 10% fetal bovine serum (Gemini, USA) for 24 h under normal cell culture conditions. Then, 5×10^4^ living G422 cells suspended in 1 μl PBS were microinjected into the right striatum of mouse brain (0.5 mm anterior and 2 mm lateral from the bregma, and 3.5 mm deep from the skull surface) as previously described^2^.

**Hematoxylin-Eosin (H&E) staining and Immunohistochemistry (IHC)**

Paraffin-embedded brain slices were used for H&E staining and IHC analysis as previously described^2^. Briefly, 4 μm-thick brain slices were deparaffinized, rehydrated, endogenous peroxidase blocked, antigen-retrieved, blocked with 5% BSA, incubated with primary and corresponding secondary antibodies (Polink-1 HRP DAB Detection System, ZSGB-BIO, China), and the colorimetric end products were produced by applying diaminobenzidine tetrachloride. Primary antibodies were anti-Ki67 (1:200, GB13030-2, Servicebio, China), anti-γ-H2AX (1:100, AP0099, ABclonal, China), anti-CD3 (1:150, ab16669, Abcam, UK), anti-PD-L1 (1:50, ABM4E54, Abcam, UK), anti-Vimentin (1:100, D21H3, CST, USA), and anti-GFAP (1:200, BA0056, BOSTER, China). Whole brain images were obtained by scanning the brain sections with an automatic slice scanning system-SV120 (Olympus, Japan). Statistical analysis used data from at least 7 slices from 5 mice brains per group.

**Whole Genome Sequencing (WGS)**

The subcutaneous G422 glioma was subject to the WGS. Library was built with >0.2 μg of DNA/tumor. The WGS was conducted on the Illumina HiSeq PE150 Sequencing System with an average sequencing depth of 28-fold by Novogene Bioinformatics Technology Co., Ltd (Beijing, China). INDEL and SNP analysis were performed with SAMtools, and their functional annotations were made by ANNOVAR. Structural variation (SV) was detected by the LUMPY software using mouse genome mm10 as reference. Raw sequence data are deposited in SRA Database (SRP276905) (https://trace.ncbi.nlm.nih.gov/Traces/sra/?run=SRR12423793).

**Bioluminescent imaging (BLI)**

For the establishment of tumor BLI *in vivo*, freshly isolated G422 cells were infected with luciferase-expressing lentivirus (Ubi-MCS-firefly-Luciferase-IRES-Puromycin, GeneChem, China) for 12 h in order to overexpress luciferase stably (G422-Luc) following the protocol provided by the manufacturer. After infection, 1×10^6^ of G422-Luc cells were subcutaneously inoculated in mice for subsequent experiments. BLI of intracranial G422-Luc tumors was performed with an animal *in vivo* optical imaging system (Spectral LagoX, USA) 10 min after a single intraperitoneal injection of 0.2 ml of sterile D-luciferin (15 mg/ml in PBS, Cayman Chemical Company, USA). The region of interest (ROI) values measured by using Amiview software (Spectral Instruments Imaging Company, USA) were used for statistical analysis of optical density values.

**Surgical treatment**

For the surgical orthotopic GBM model, G422 cells were inoculated into a superficial site of the right cerebrum (0.5 mm anterior and 2 mm lateral from the bregma, and 2 mm deep from the skull surface) of mice. On day 7 after G422 cell inoculation, intracranial tumor burden was confirmed by BLI. Next, a skull cap of 5 mm in diameter with the original injection site in the center was removed to fully expose the tumor. Within 20 min post the skull cap removal, the tumor mass was macroscopically completely removed with micro-forceps under a stereo microscope until the white walls, which indicates the normal brain parenchyma, were macroscopically apparent in the surgical cavity^3^. Hemostasis was accomplished by using gel foam. Complete removal of tumors was verified by BLI 24 h after the surgery. Less than 5% surgical death occurred within 48 h after the surgery.

**Radiotherapy (RT), Temozolomide (TMZ) and RT/TMZ**

Mice in their prone position were irradiated with an X-ray irradiator (RS-2000 pro, Rad Source Technologies, USA) under fixed parameters (160 KV, 25 mA, 1 Gy/48.4 sec). Lethal effects of the total body irradiation (TBI) on normal adult Kunming mice were recorded after a single dose of irradiation (2, 4, 5, 6, 8 or 10 Gy) (Figure S3). No death was found in normal adult Kunming mice subjected to whole brain irradiation (WBI) with the maximal dose (10 Gy) that was used in the following WBI experiments. During WBI, a reflector was used to improve the uniformity of irradiation and the other parts of the mouse body were blocked from irradiation with a 3 mm lead plate. Temozolomide (AbMole, China) was dissolved in 0.5% CMC-Na at a final concentration of 5 mg/ml. It was administered daily to the mice with the dose of 50 μg TMZ per gram of body weight via oral gavage for five consecutive days, followed by a 2-day interval and another 5-day same TMZ regimen (Figure 2D). For the RT/TMZ, two 5 Gy irradiations, interrupted by a 5-minute interval, were performed on the first day of the TMZ administration.

**Mannose and Molidustat treatment**

For mannose/glucose (AbMole, China) treatment, each mouse received a total amount of 40 g mannose or glucose, dissolved in 200 ml sterile water (20% (w/v)), per week via normal drinking. In addition, each mouse was supplemented with one dose of 40 mg mannose or glucose (200 μl of 20% (w/v) mannose or glucose solution) by oral gavage three times per week^4^ (Figure S5). For the RT/TMZ/Man therapy, mannose and TMZ administrations were started on the same day (Figure S6). Molidustat (Selleck Chemicals, China) was dissolved in a solvent composed of ethanol/solutol HS 15/water (10/20/70, v/v/v) with a final concentration of 0.5 mg/ml^5^. It was administered with a dose of 5 μg per gram of body weight daily via oral gavage on the same day of TMZ administration (Figure S10).

**Metformin and disulfiram/copper gluconate treatments**

Metformin (MET, AbMole, China) or disulfiram (DSF, AbMole, China)/copper gluconate (CuGlu, AbMole, China) was delivered on the same days of the TMZ administration (Figure S7). Metformin was administered with a dose of 400 μg/gram of body weight per day via oral gavage. For the disulfiram/copper gluconate (DSF/Cu) regimen, DSF and CuGlu were delivered separately. DSF was dissolved in a solvent composed of ddH_2_O/cremophor/DMSO/ethanol (7.5/1.5/0.5/0.5, v/v/v/v) with a final concentration of 10 mg/ml. It was administered via oral gavage with a dose of 100 μg/gram of body weight per day. CuGlu was administered also via oral gavage with a dose of 2 μg/gram of body weight per day.

**Positron Emission Tomography (PET)/Computerized Tomograph (CT) Imaging**

Mice were subject to PET/CT combined imaging on the 7^th^ day following orthotopic or subcutaneous injection of G422 cells. After a 12 h-fasting, each mouse received 200 μl of 20% mannose by oral gavage. Twenty minutes later, 2-deoxy-2-[18F]fluoro-D-glucose (^[18F]^FDG) (167.5 ± 3.80 μci) in 200 μl of normal saline were administered via tail vein. After uptake phases of 30 min for ^[18F]^FDG, PET/CT scans were sequentially performed 10 min by using the Inliview 3000B animal PET/SPECT/CT system (Beijing Novel Medical Equipment Co., Ltd, China) and the data were analyzed by using PMODE software.

**Targeted Metabolomics by** [**Liquid Chromatography-Mass Spectrometry (LC-MS)**](https://www.baidu.com/link?url=mp1zpTmODjGFf1JCZicjcW0XE-kF0_JZoWDIzNV13ILqHRJAEx9NX3OisPBNyGdEvql45_kfKB4JDQ8i9ExGQK&wd=&eqid=d7cb472d00050ce5000000065ea6842b)

Adult mice bearing subcutaneous G422 tumors of 0.8-1 cm in diameter were treated with either mannose (Man: 20% mannose in drinking water + 40 mg mannose via oral gavage), RT (10 Gy, total body irradiation, one dosage) plus TMZ (50 μg/gram of body weight/day), or RT/TMZ/Man for 2 days and then the tumors were isolated for metabolomics. More than 200 mg of tumor tissues were used for the measurement of targeted metabolites by LC-MS analysis (Novogene Bioinformatics Technology Co., Ltd) and the results were analyzed by using SCIEX OS V1.4 software for chromatographic peak analysis. Differentially expressed metabolites were screened by the following criteria: VIP>1.0, the difference multiple FC>1.2 or FC<0.833 and *P*value<0.05. With *P*value≤0.05 as the threshold, the KEGG entry that meets this condition was defined as significant enriched KEGG entry in the differential metabolites.

**Western blotting analysis**

Adult Kunming male mice bearing subcutaneous G422 tumors of 0.8-1 cm in diameter were treated with either Mannose (Man: 20% mannose in drinking water + 40 mg mannose via oral gavage), RT (10 Gy, TBI, one dosage) plus TMZ (50 μg/gram of body weight/day), or RT/TMZ/Man for 2 days and then the subcutaneous tumor tissues were isolated for Western blotting analyses (n=5/group). Briefly, G422 tumor mass tissue cell lysates were collected and dispersed in radioimmunoprecipitation assay lysis buffer containing phenylmethanesulfonyl fluoride. After acrylamide gel electrophoresis, proteins were transferred to the PVDF membrane, which were first incubated with primary antibody, followed by the corresponding near-infrared fluorescent secondary antibody. Primary antibodies included anti-HIF-1α (1:2000, H1alpha67, Abcam, UK), anti-VEGF (1:2000, BA0407, BOSTER, China), anti-PHD2 (1:2000, GB111030, Servicebio, China) and anti-β-tubulin (1:2000, 10094-1-AP, Proteintech, China). Following antibody incubation, intensity of each protein band on the PVDF membrane was visualized and quantified using the Odyssey Infrared Imaging System (LI-COR Bioscience, USA).

**Statistical analysis**

Animal survivals were analyzed by the Kaplan-Meier estimate and compared using a log-rank (Mantel Cox) test. Two-tailed unpaired t-test was used to analyze the differences between two unpaired groups. Paired t-test was used to analyze two paired groups. One-way analysis of variance (ANOVA) with Dunnett’s test was used to compare one-factor variable experiments among multiple groups. All values were reported as mean ± SEM. Differences were considered significant at a value of *P*<0.05. All analyses were performed using GraphPad Prism 7.

**Figure S1**

**
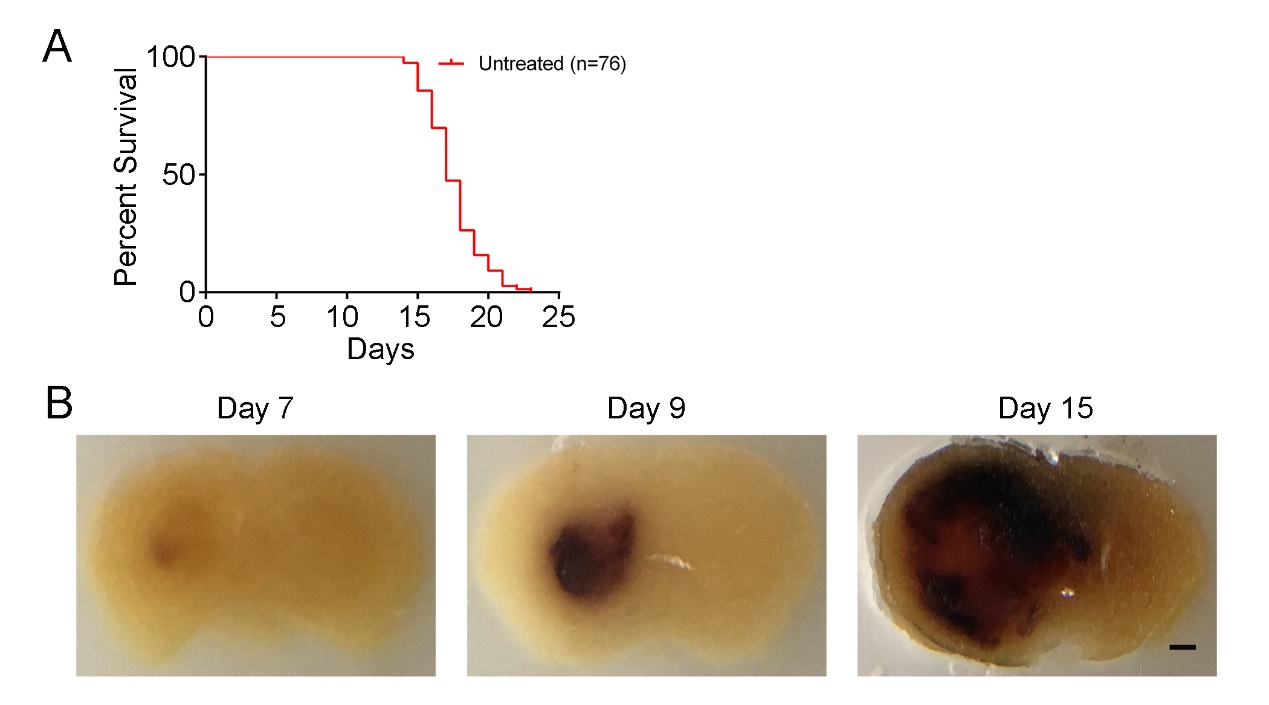
**

**Figure S1** Survival time and gross pathology of an orthotopic murine triple-negative primary GBM model. A, The Kaplan-Meier survival of 76 untreated mice intracranially injected with 5×10^4^ G422 cells pooled from 10 individual experiments in this study. All G422-mice died between 14-23 days post implantation (*p.i.*) and exhibited a narrowly ranged median survivals of 16-19 days. B, Representative images showing hemorrhage in the paraffin embedded G422 gliomas collected on day 7, 9 and 15 *p.i.*. Scale bar, 50 μm.

**Figure S2**

**
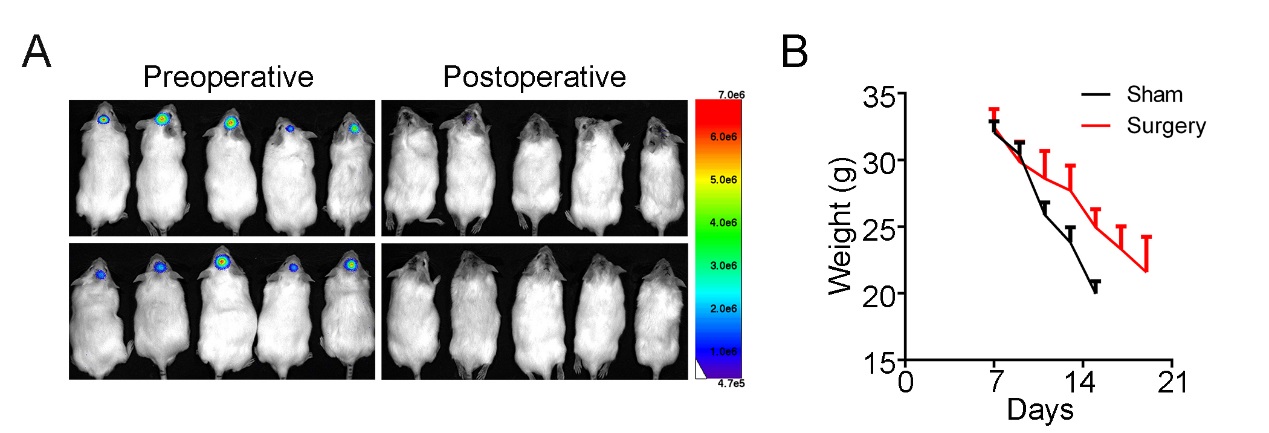
**

**Figure S2** Therapeutic responses of the G422^TN^-GBM model to the conventional surgical therapy. A, Representative luciferase bioluminescent images of the intracranial tumors before and after surgery. B, Body weight changes over time of the G422-mice with or without receiving surgery (n=8/group).

**Figure S3**

**
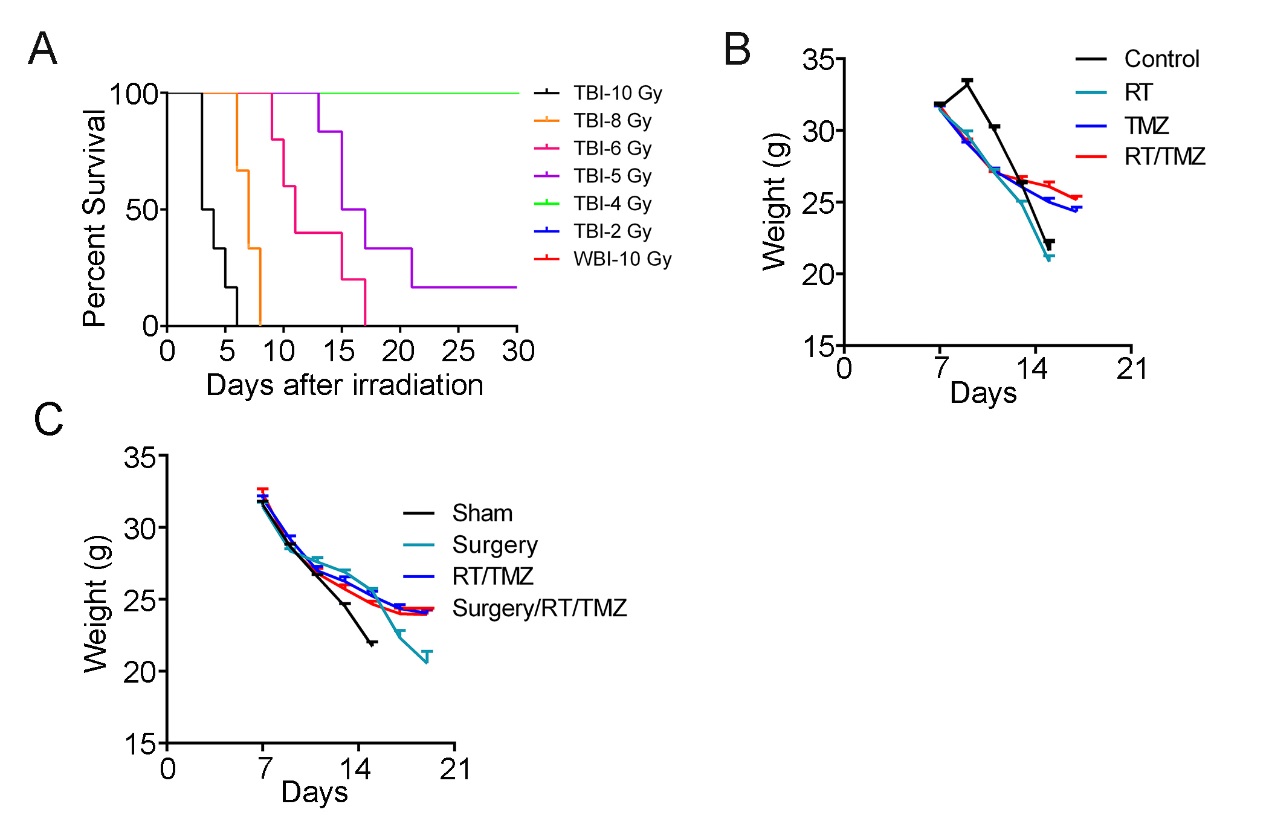
**

**Figure S3** Therapeutic responses of the G422^TN^-GBM model to the conventional Surgery/RT/TMZ therapy. A, The Kaplan-Meier survivals of normal mice that received a single exposure of irradiation with the indicated dosage (n=6/group). TBI, total body irradiation; WBI, whole brain irradiation. B, Body weight changes over time of the G422-mice subjected to different treatments on day 7 *p.i.* as indicated (n=7/group). C, Body weight changes over time of the G422-mice subjected to the indicated treatments. RT/TMZ, TMZ concurrent radiotherapy; Surgery/RT/TMZ, RT/TMZ preceded by surgery (n=8/group).

**Figure S4**


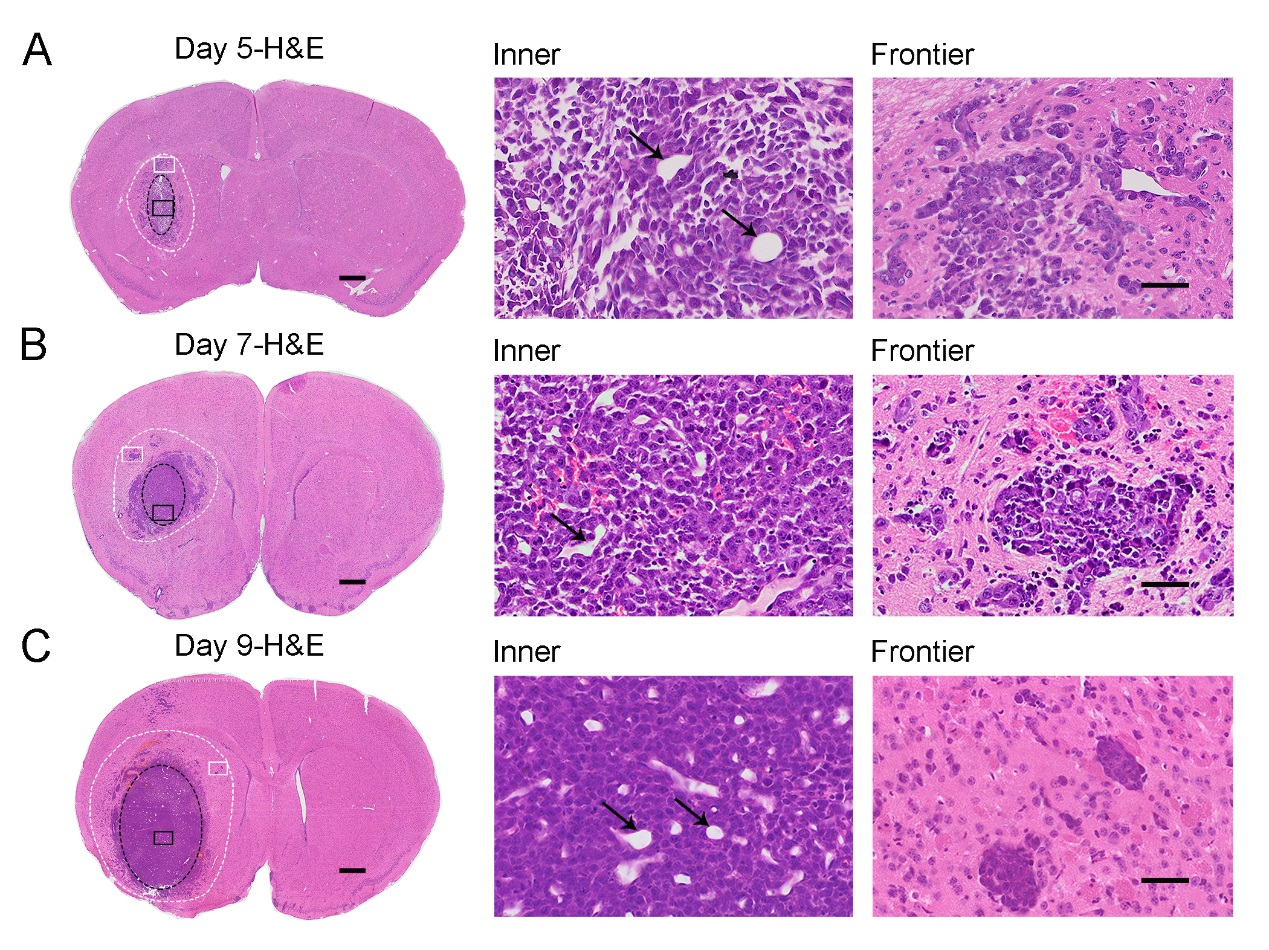


**Figure S4** H&E staining shows progressive growth of G422 tumors on day 5, 7, and 9 *p.i.*. A-B-C, Representative H&E staining of the G422 glioma on day 5 (A), 7 (B) and 9 (C) *p.i.*. The black dotted ellipse delineates the boundary of tumor parenchyma while the outer irregular white dotted circle delineates the infiltration boundary. Left panels show the images of the whole brain section, while the middle and right panels show the amplified inner and frontier tumor areas marked by the black or white rectangles, respectively, in the left panels. Arrows indicate microvessels inside the tumor. Scale bar, 50 μm.

**Figure S5**

**
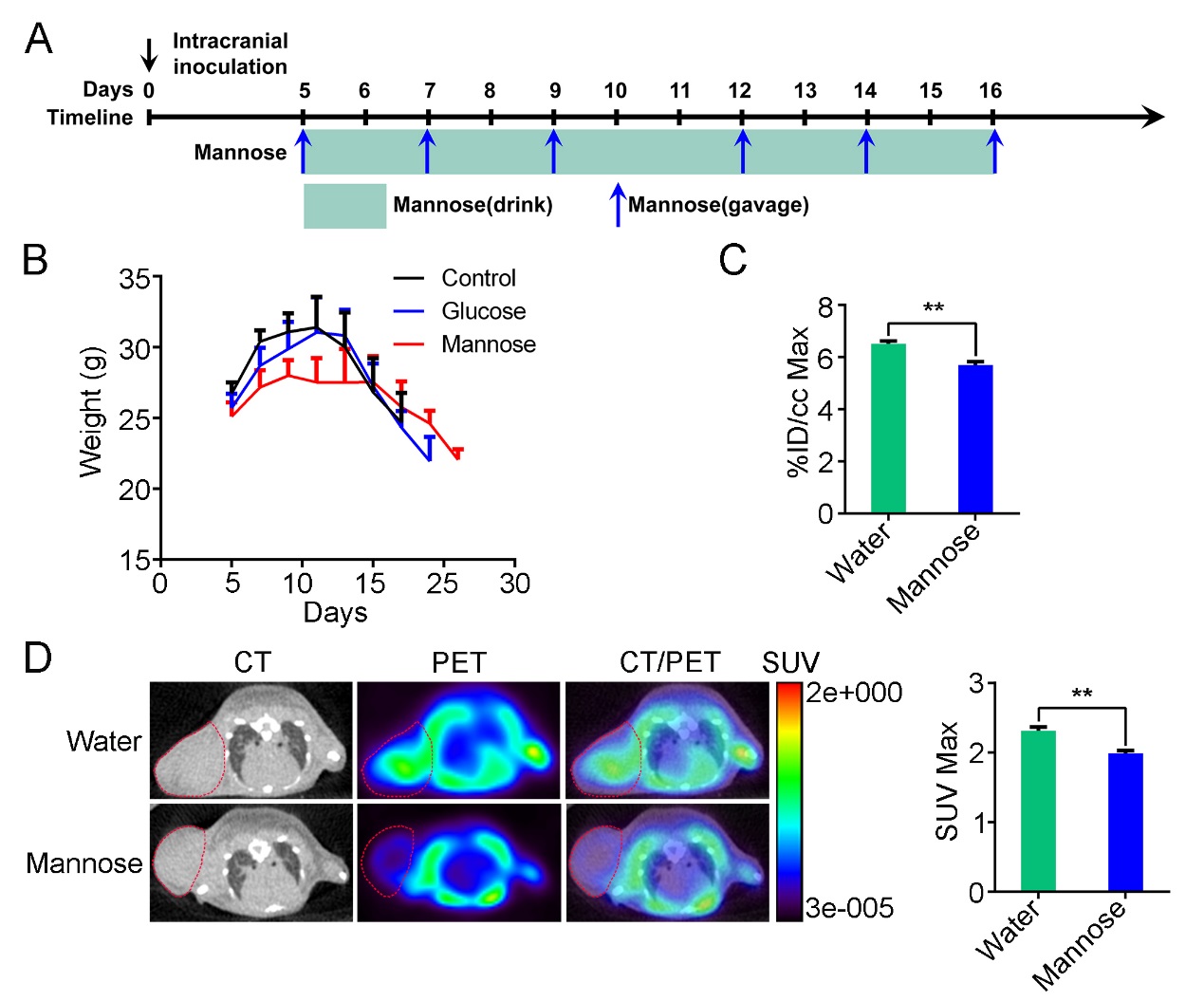
**

**Figure S5** Mannose monotherapy enhances survival of the G422-mice by interfering with glucose metabolism. A, Schematic diagram of mannose administration started on day 5 *p.i.* for Figure 3B. B, Body weight changes over time of the G422-mice after treated with mannose or glucose in Figure 3B (Control: regular water, n=7; Mannose or Glucose group: n=8). C, Statistical analysis of the %ID/cc Max compared between intracranial G422 gliomas treated with water and mannose in Figure 3A (n=5/group). D, Left, representative PET/CT imaging of subcutaneous G422 gliomas denoted by the red dotted circles. Right, statistical analysis of the SUV Max compared between tumors treated with water and mannose (n=5/group). (** *P*<0.01)

**Figure S6**


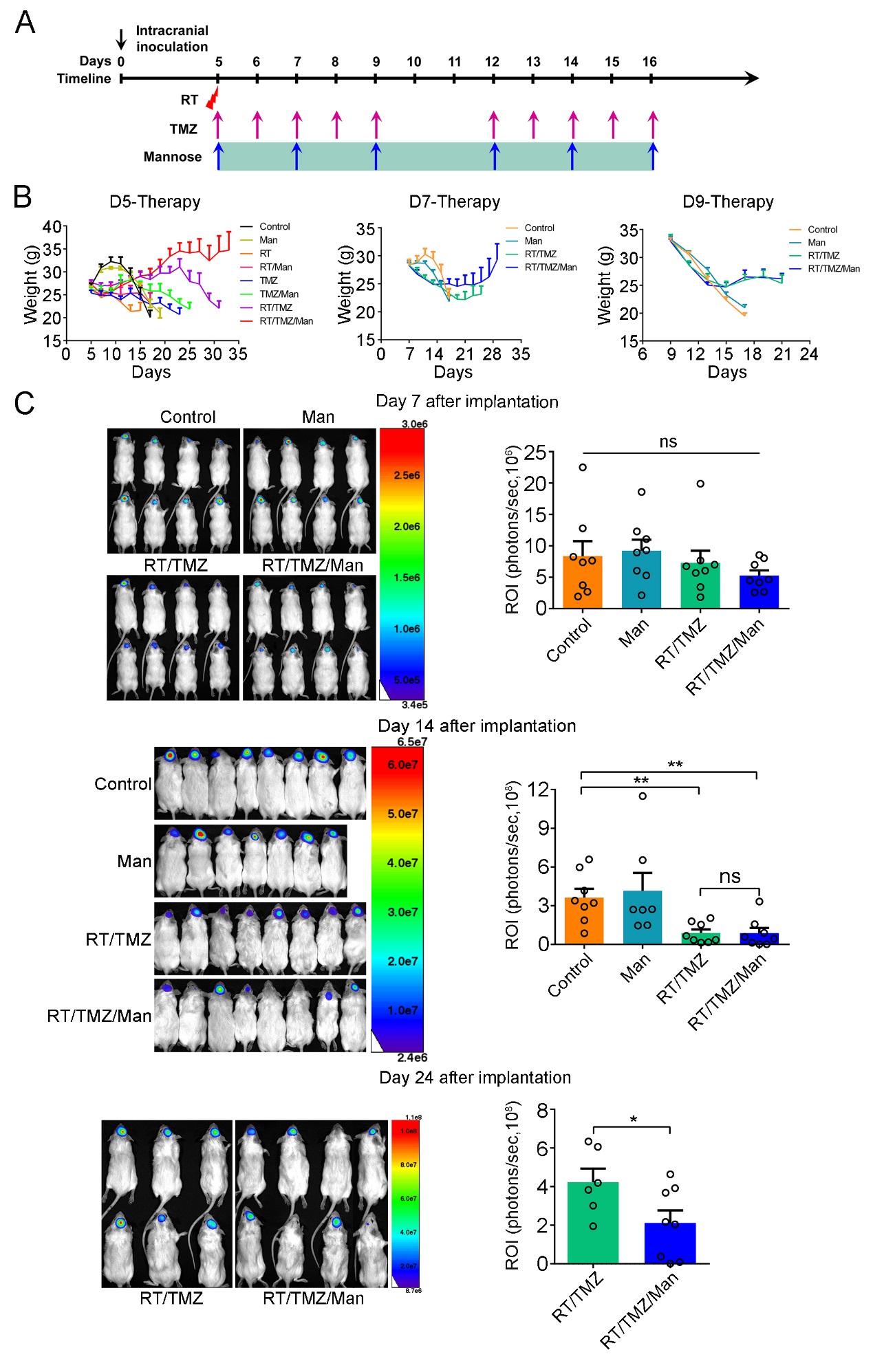


**Figure S6** Mannose plus RT/TMZ achieves long-term survival in the G422-mice. A, Schematic diagram of the combined mannose plus RT/TMZ (RT/TMZ/Man) started on day 5 *p.i.*. B, Body weight changes over time of the G422-mice treated with the indicated therapies started on day 5 (D5-Therapy), 7 (D7-Therapy) or 9 (D9-Therapy) *p.i.*. C, Serial measurements of the luciferase-luminescent intensity of tumors from the G422-mice in D7-Therapy, performed on day 7, 14 and 24 (n=6-8/group, * *P*<0.05; ** *P*<0.01; ns, not statistically significant).

**Figure S7**


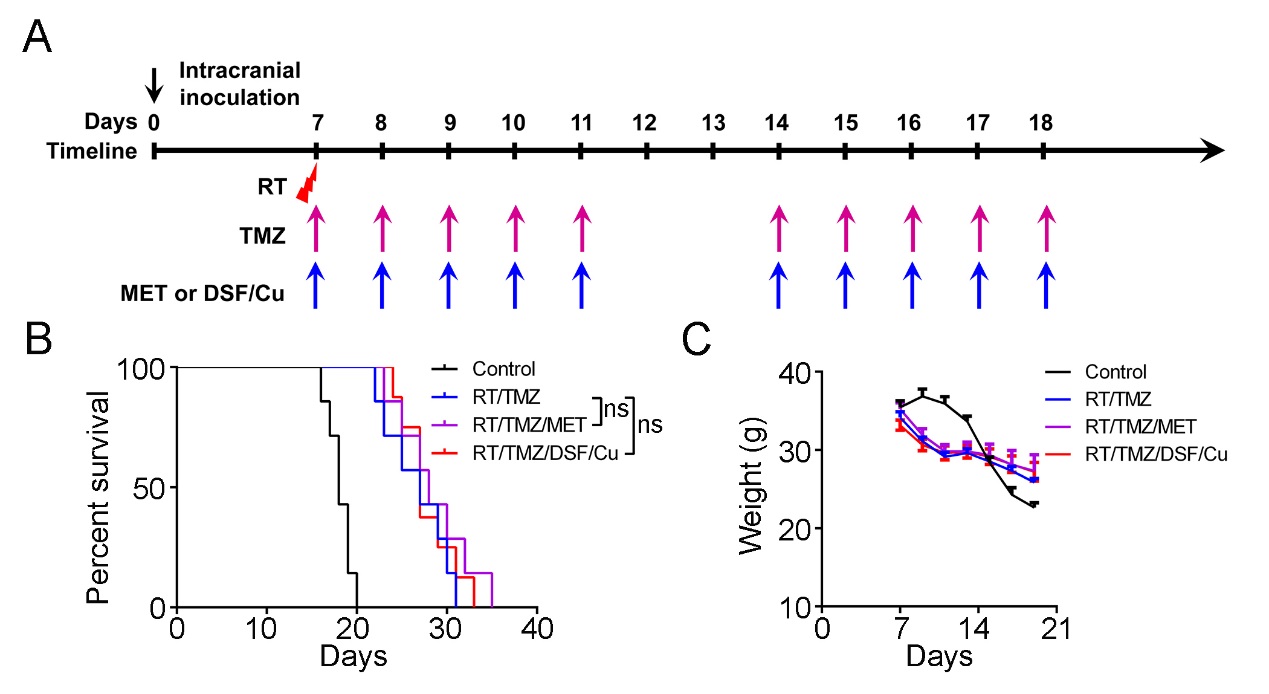


**Figure S7** Effects of RT/TMZ in combination with metformin or disulfiram/copper gluconate in G422-mice. A, Schematic diagram depicting RT/TMZ plus metformin (RT/TMZ/MET) or disulfiram/copper gluconate (RT/TMZ/DSF/Cu) regimens started on day 7 *p.i.*. B, The Kaplan-Meier survivals of the G422-mice subjected to the indicated treatments started on day 7 *p.i.* (n=7-8/group, ns, not statistically significant). C, Body weight changes over time of the same groups of the G422-mice in (B) that have subjected to the indicated treatments started on day 7 *p.i.*.

**Figure S8**

**
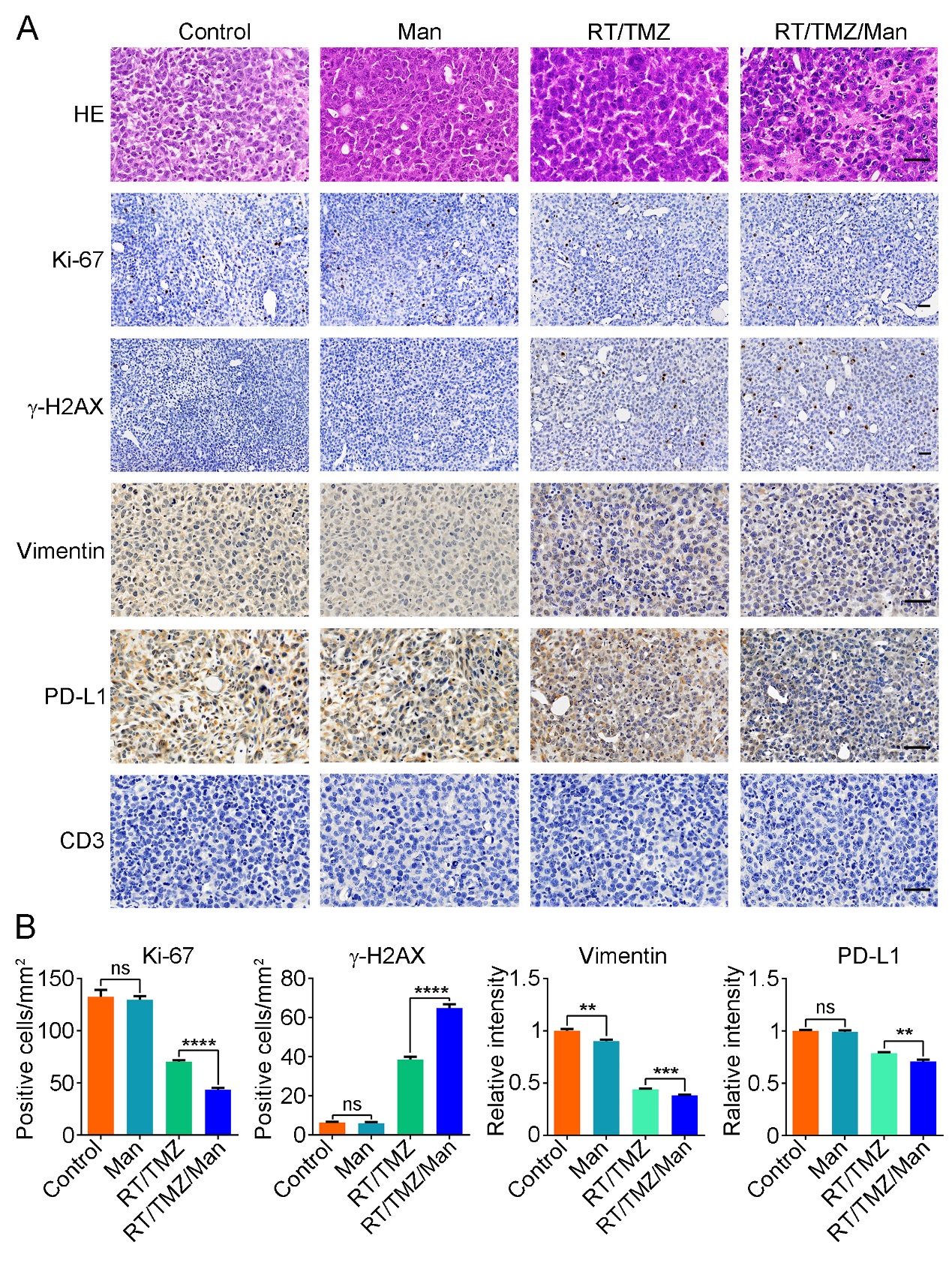
**

**Figure S8** Mannose exerts synergistic antitumor effect via inhibiting tumor proliferation, invasion and enhancing DNA damage. A, Representative micrographs of H&E stained G422-gliomas from mice that received Man, RT/TMZ or RT/TMZ/Man treatments for two days on day 7 *p.i.*. Sectioning from the same tumors were also subjected to IHC showing the expression of Ki-67, γ-H2AX, Vimentin, PD-L1 and CD3. Scale bar, 50 μm. B, Statistical analysis of the Ki-67, γ-H2AX, Vimentin and PD-L1 expression in G422-gliomas from mice that received Man, RT/TMZ or RT/TMZ/Man treatments for two days on day 7 *p.i.*. Error bar indicates standard error of mean. (n=5/group, ** *P*<0.01; *** *P*<0.001; **** *P*<0.0001; ns, not significant)

**Figure S9**

**
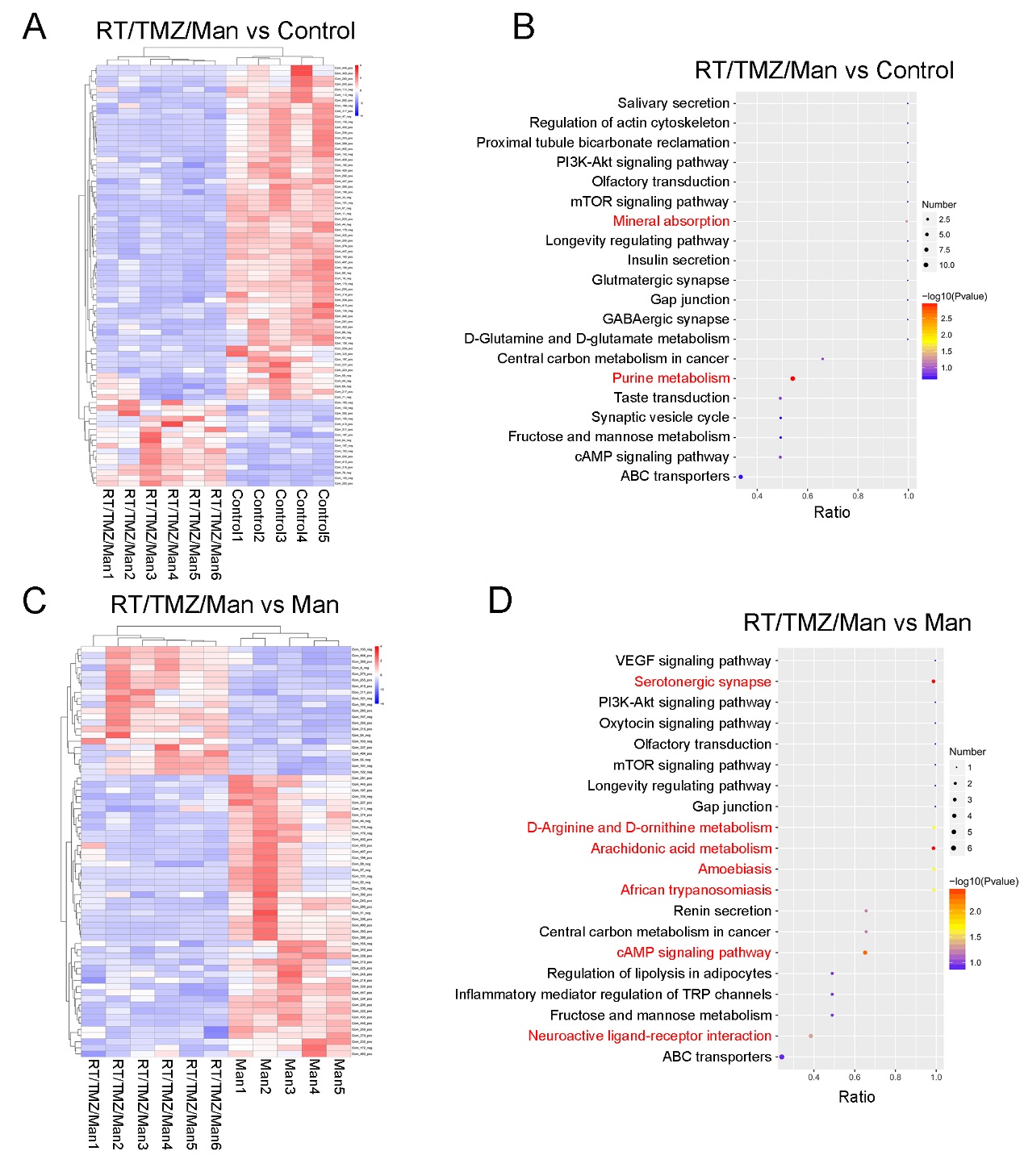
**

**Figure S9** Metabolomic screening identifies the HIF-1 signaling pathway as a key mechanism underlying the efficacy of the RT/TMZ/Man regimen. A-C, Clustered metabolite heat map showing significantly differential metabolites between the RT/TMZ/Man and the Control (A) or Man (C) (n=5-6/group). B-D, The top 20 differential pathways between the RT/TMZ/Man and the Control (B) or Man (D) identified by the KEGG pathway enrichment analysis. Significantly altered pathways in (B), (D) are highlighted in red fonts.

**Figure S10**


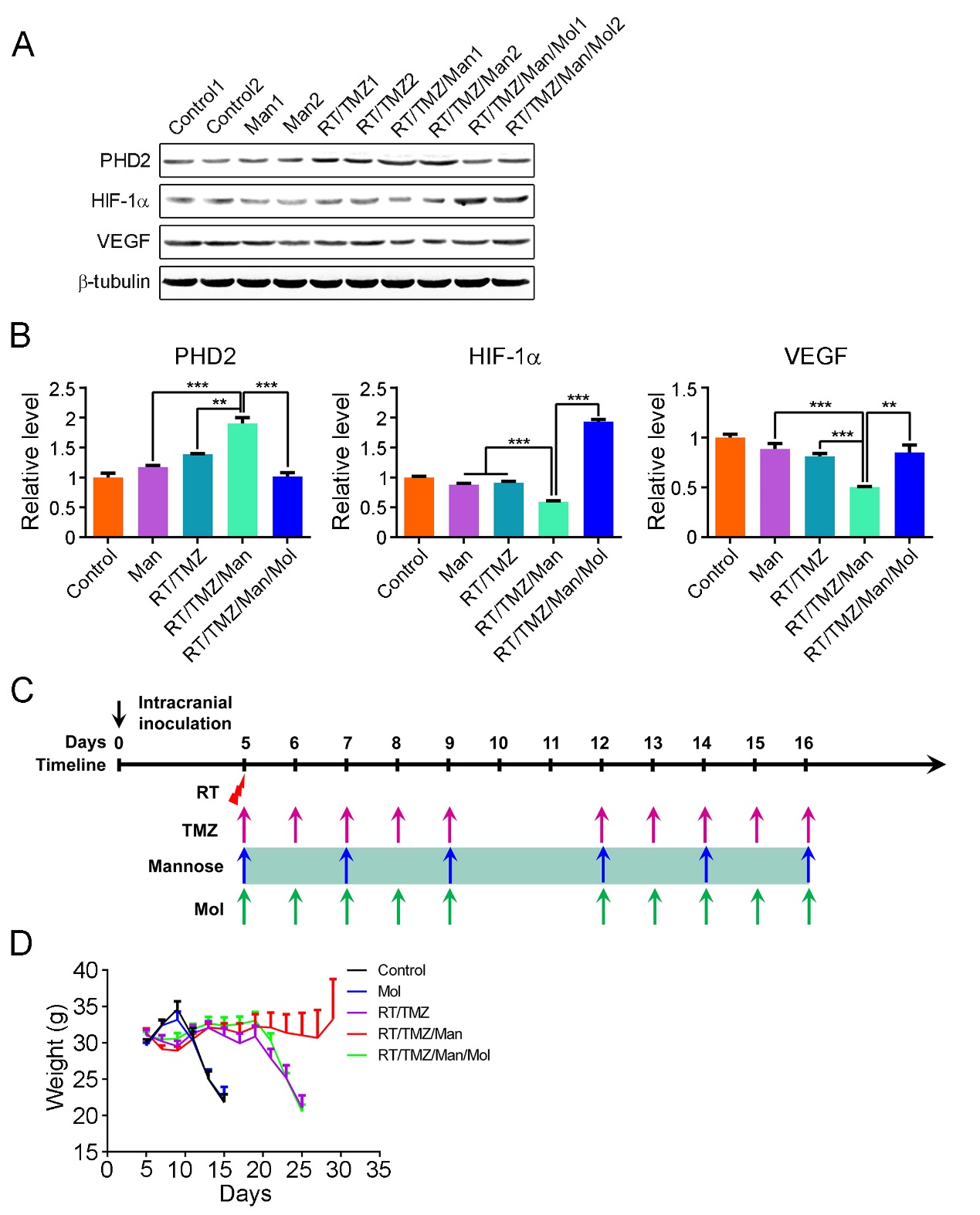


**Figure S10** Mannose exerts synergistic antitumor effect by targeting PHD2/HIF1-α/VEGF signaling pathway. A, Representative Western blots showing the protein levels of PHD2, HIF-1α and VEGF in G422 tumors from mice treated with the Man, RT/TMZ, RT/TMZ/Man or RT/TMZ/Man plus Molidustat (RT/TMZ/Man/Mol). Subcutaneous G422 tumors at 1 cm in diameter are subjected to Man, RT/TMZ, RT/TMZ/Man or RT/TMZ/Man/Mol treatments for two days. Then, the tumors are isolated for Western blotting analysis with specific PHD2, HIF-1α and VEGF antibodies. The results showed that the expression levels of PHD2 are evidently increased in RT/TMZ/Man compared to RT/TMZ and Man groups, but reduced after the addition of Molidustat (RT/TMZ/Man/Mol). The expression levels of HIF-1α and VEGF show opposite changes of PHD2 upon RT/TMZ/Man or RT/TMZ/Man/Mol treatments. B, Statistical analysis of the protein levels of PHD2, HIF-1α and VEGF in G422 tumors from mice treated with the Man, RT/TMZ, RT/TMZ/Man or RT/TMZ/Man/Mol. Error bar indicates standard error of mean (** *P*<0.01; *** *P*<0.001; n=5/group). C, Schematic diagram of the RT/TMZ/Man combined with Molidustat (Mol), RT/TMZ/Man/Mol, regimen started on day 5 *p.i.*. D, Body weight changes over time of the G422-mice treated with the indicated therapies started on day 5 *p.i.*.

**REFERENCES**

1. Chen B H, Shao W Z, Zhu G C, et al. Transplantable mouse glioblastoma and its morphologic observation. Chin Med J (Engl). 1983; 96(2):111-6.

2. Chen R Q, Xu X H, Liu F, et al. The Binding of PD-L1 and Akt Facilitates Glioma Cell Invasion Upon Starvation via Akt/Autophagy/F-Actin Signaling. Front Oncol. 2019; 9:1347.

3. Xue J, Zhao Z, Zhang L, et al. Neutrophil-mediated anticancer drug delivery for suppression of postoperative malignant glioma recurrence. Nat Nanotechnol. 2017; 12(7):692-700.

4. Gonzalez P S, O'Prey J, Cardaci S, et al. Mannose impairs tumour growth and enhances chemotherapy. Nature. 2018; 563(7733):719-723.

5. Flamme I, Oehme F, Ellinghaus P, et al. Mimicking hypoxia to treat anemia: HIF-stabilizer BAY 85-3934 (Molidustat) stimulates erythropoietin production without hypertensive effects. PLoS One. 2014; 9(11):e111838.
